# Translation, cross-cultural adaptation, and validation into Brazilian Portuguese of the Functional Rhinoplasty Outcome Inventory-17 (FROI-17) for use in patients undergoing Functional Rhinoplasty^☆^

**DOI:** 10.1016/j.bjorl.2026.101773

**Published:** 2026-05-23

**Authors:** Maria Eduarda Malosá Jorge, Ana Caroline Soares Silva, Araken Quedas, Daniela Malheiros, Guilherme Mororo, Ingo Baumann, Eduardo Landini Lutaifi Dolci, Cinthya Cosme Gutierrez Duran

**Affiliations:** a Universidade Nove de Julho (UNINOVE), Biophotonics Medicine Postgraduate Program, São Paulo, SP, Brazil; b Irmandade da Santa Casa de Misericórdia de São Paulo, Department of Otorhinolaryngology, São Paulo, SP, Brazil; c Heidelberg University Hospital, Vice Chairman, Department of Otolaryngology, Heidelberg, Germany

**Keywords:** Rhinoplasty, Quality of life, Surveys and questionnaires, Validation study, Cross-cultural comparison, Nasal obstruction

## Abstract

**Objectives:**

To translate, culturally adapt into Brazilian Portuguese, and validate the Functional Rhinoplasty Outcome Inventory-17 (FROI-17) questionnaire for quality-of-life assessment in patients undergoing functional rhinoseptoplasty.

**Methods:**

A validation study was conducted following the Guidelines for the Process of Cross-Cultural Adaptation of Self-Report Measures and the COSMIN checklist. The translated FROI-17 was applied to adult patients undergoing rhinoseptoplasty. The following measurement properties were assessed: internal consistency (Cronbach’s α), agreement (Standard Error of Measurement and Minimal Detectable Difference), intra-rater reliability (Intraclass Correlation Coefficient, ICC), and convergent validity (correlation with SF-36 and SCHNOS).

**Results:**

The Brazilian version of the FROI-17 showed high internal consistency (Cronbach’s α = 0.93) and excellent intra-rater reliability (ICC = 0.95). Agreement measures were within acceptable clinical thresholds. Convergent validity was confirmed by moderate to strong correlations with SF-36 and SCHNOS. No statistically significant differences were observed between sexes in total FROI-17 scores (p = 0.220), although women presented greater score variability.

**Conclusion:**

The Brazilian Portuguese version of the FROI-17 proved to be a valid and reliable instrument for assessing functional and aesthetic outcomes in patients undergoing rhinoseptoplasty. It enables objective, standardized evaluation of surgical results and enhances clinical decision-making in otorhinolaryngology.

**Evidence-based medicine:**

Levels of evidence 3.

## Highlights

• Translation, adaptation, and validation of the FROI-17 for Brazilian Portuguese.
• Process followed COSMIN and cross-cultural adaptation guidelines ensuring equivalence.
• Validity supported by moderate to strong correlations with SF-36 and SCHNOS scores.

## Introduction

The assessment of Health-Related Quality of Life (HRQoL) has become an essential parameter in evaluating therapeutic outcomes, especially in procedures such as rhinoseptoplasty, which affect both functional and aesthetic aspects of the nasal structure. In addition to objective measures of clinical improvement, it is increasingly necessary to demonstrate measurable gains in patient-perceived quality of life.^1^^,^^2^

To ensure accurate and comparable results across cultures and populations, the use of validated and culturally adapted instruments is imperative. The process of cross-cultural adaptation must follow standardized guidelines to ensure conceptual, semantic, and measurement equivalence.^2^^,^^3^ In Brazil, although generic instruments such as the SF-36 have been validated for Portuguese,^4^ there remains a gap regarding disease-specific tools for patients undergoing rhinoseptoplasty.

The Functional Rhinoplasty Outcome Inventory-17 (FROI-17) is a questionnaire developed to assess nasal function-related quality of life, incorporating both functional limitations and aesthetic concerns.^5^ Its validity and reliability have been demonstrated in European populations, and it has been increasingly used as an outcome measure in clinical studies.^6^^,^^7^ However, no version adapted to Brazilian Portuguese was available until now.

Given the increasing demand for rhinoplasty in Brazil, especially for aesthetic purposes, the availability of a validated tool in Portuguese is essential. Such a tool would allow for standardized evaluation of outcomes, support evidence-based surgical decisions, and improve the identification of patients more likely to benefit from intervention.^8^

The aim of this study was to translate, cross-culturally adapt, and validate the Functional Rhinoplasty Outcome Inventory-17 (FROI-17) into Brazilian Portuguese for use in adult patients undergoing rhinoseptoplasty.

## Methods

This was a single-center, prospective validation study conducted at the Department of Otolaryngology of the Irmandade da Santa Casa de Misericórdia de São Paulo, in São Paulo, Brazil. The study was designed to assess the cross-cultural adaptation and measurement properties of a patient-reported outcome instrument. All procedures were approved by the Human Research Ethics Committee of the Irmandade da Santa Casa de Misericórdia de São Paulo (approval number: 74191823.1.0000.5479). Participants received verbal and written explanations regarding the study's objectives, risks, and procedures, and were included only after signing the informed consent form.

### Participants

Participants were eligible for inclusion if they were adults aged 18 years or older and were undergoing or had undergone rhinoseptoplasty. Recruitment was conducted via telephone contact. Exclusion criteria included any cognitive or language impairment that interfered with the comprehension of the questionnaires, or refusal to complete any of the assessment instruments.

All questionnaires were administered by a single trained interviewer, a medical student under the supervision of physicians from the Department of Otolaryngology.

### Study design

This study was designed to analyze the measurement properties and conduct the cross-cultural adaptation of a patient-reported outcome measure. Three instruments were used: the Short Form Health Survey (SF-36) and the Standardized Cosmesis and Health Nasal Outcomes Survey (SCHNOS) ‒ both previously translated and validated for Brazilian Portuguese ‒ and the Functional Rhinoplasty Outcome Inventory-17 (FROI-17).

Authorization to translate and validate the FROI-17 into Brazilian Portuguese was granted by its original authors.^5^ The translation and cultural adaptation process followed the internationally accepted guidelines proposed by Beaton et al.,^3^ ensuring semantic, idiomatic, experiential, and conceptual equivalence between the original and translated versions.

### Translation and adaptation

The translation process consisted of six phases: “Phase I” – initial translation; “Phase II” – synthesis of translations; “Phase III” – reverse translation; “Phase IV” – committee of experts; “Phase V” – pre-testing of the final version; and “Phase VI” – submission and consideration of all written reports.^9^

“Phase I” – Initial translation

“Phase I” consists of translating the questionnaire in its original English version into Portuguese Brazilian. For this stage, two translators (“TR1” and “TR2”) were invited to translate the questionnaire independently, without any type of communication or contact between them.

It was ensured that none of the translators had had previous contact specifically with the questionnaire to be translated, ensuring that they did not have prior knowledge of the content. The translators were native speakers of English and fluent in Portuguese Brazilian. “TR1” had previous knowledge of concepts and terms used in the health area, to provide a possibly more accurate translated version in the language of technical terms in the area. The “TR2”, on the other hand, had only knowledge of lay people about themes related to the health area without knowledge of technical terms.

The two versions translated into Portuguese Brazilian (T1 and T2) contained comments by the translators so that doubts and possible discrepancies could be discussed in “Phase II”.

“Phase II” – Synthesis of translations

“Phase II” consists of the synthesis of the translations (“T1” and “T2”) performed in “Phase I”. Under the coordination of the principal investigator, the translators met to produce a unified version (“T1‒2”), in which any discrepancies were discussed so that there was a consensus among the translators. The synthesis process was documented in a report so that the adjustment stage was reported with its proper justifications.

“Phase III” – Reverse translation

In “Phase III”, a third Translator (“TR3”), native in the Portuguese Brazilian language and fluent in the English language, who did not know the original questionnaire, back-translated the unified version (“T1‒2”) in Portuguese-Brazilian into English (“RT1-2”). The retro-translated version (“RT1‒2”) was sent to the original author and study team of the questionnaire for proper validation of the content. The original author and study team identified discrete items in which the content of the back translation did not meet the intended meaning of the original items. Based on this feedback, modifications in the content of the “RT1‒2” version, and “Phase II” was resumed so that the syntheses of the signaled points could be discussed and modified.

“Phase IV” – Expert committee

In “Phase IV”, the reverse-translated version (“RT1‒2”) was analyzed by a committee of experts to perform the cross-cultural equivalence of the questionnaire. This committee was composed of 4 professionals: 2 health professionals, 1 language professional, 1 methodologist and the translators involved in the process of “Phases I and II” (“TR1”, “TR2” and “TR3”). The authors of the original questionnaire were in contact with this committee during this process.

With the availability of the original questionnaire and the translations performed and their respective reports with their observations, the objective of this committee was to review the translations, remedy any discrepancies found between them and develop the pre-final version for field testing. This committee aimed to achieve equivalence from the original language to the translated version in the following areas:

Semantic equivalence – Assessed grammatical and vocabulary equivalence by comparing whether words have the same meaning during translation.

Idiomatic equivalence – Dealt with the difficulties in translating colloquial expressions from the English language.

Experiential or cultural equivalence – Sought to equate the terms used with the lived experience of the population to which it is intended.

Conceptual equivalence – Adapted the concepts of terms used between both languages.

“Phase V” – Submission and appraisal

In “Phase 5”, an audit of the entire process was carried out, where all reports and translated versions were presented. The original questionnaire study team (CD and IB) reviewed all phases to ensure that they were properly followed and that they were in accordance with the process.

### Measurement properties

Evaluations related to measures or outcomes of an instrument were performed. For statistical analysis, the SPSS software for Windows (version 22; SPSS inc.; USA, Armonk, NY). The Shapiro-Wilk test was used to determine the normality of the data. Parametric variables were described as mean and Standard Deviation (SD). Categorical variables were described as absolute numbers, percentage, and frequency. Clinimetric analyses were performed considering internal consistency, agreement, intra-rater reliability, convergent validity, and ceiling-floor effect.

### Sample size

For the sample calculation, we followed the recommendations of the guideline of *the* “COnsensus-based Standards for the selection of health Measurement Instruments” (COSMIN), which suggests that studies with the objective of performing an adequate evaluation of internal consistency, agreement, reliability and construct validity, the sample size can be classified as being considered an “adequate” sample when composed of 100 or more participants; a sample “good” when composed of between 50 and 99 participants; “moderate” when composed of between 30 and 49 participants; and a “small” sample when composed of fewer than 30 participants.

## Results

### Sample characteristics

A total of 61 candidates were eligible to participate in the present study. Of these, 50 met the inclusion criteria. Ten candidates refused to answer the questionnaires, and one provided incomplete responses.

The mean age of participants was 50.20-years (SD = 15.07), with 56% identifying as female and 44% as male (Table 1).

**Table 1:** Demographic and clinical characteristics of the sample.

Variables	(n = 50)
Anthropometric	Mean (SD) / N (%)
Age (years)	50.20 (15.07)
Gender	N (%)
Female	28 (56%)
Male	22 (44%)
Race	
White, non-Hispanic	34 (68%)
Asian	1 (2%)

SD, Standard Deviation; %, Percent.

### Internal consistency and reliability

Test-retest analysis showed that both reliability and internal consistency were adequate. Cronbach’s alpha was 0.93, indicating excellent internal consistency. Intraclass Correlation Coefficient (ICC) was 0.95, which is also considered excellent. These evaluations were performed for each domain of the questionnaire.

### Agreement

The Standard Error of Measurement (SEM), which reflects the measurement error, was calculated as: SEM=SD×√(1-ICC). The Minimal Meaningful Difference (MMD) represents the smallest change that can be interpreted as a real difference. It was calculated using the formula: MMD=1.96×√2×SEM. MMD was also expressed as a percentage (MMD%), providing an estimate of the relative random measurement error. The percentage was calculated as: $MMD\%\;=\;\left(MMD-mean\;total\;score\right)\times \;100;$ a minimum detectable change percentage <30% was considered acceptable, and <10% excellent (Table 2, Table 3).

**Table 2:** FROI Reproducibility analysis.

	Test Mean (SD)	Retest Mean (SD)	Internal Consistency	Agreement		Confiability	Validity
			*α Croncach*	SEM	MMD90	ICC (95%)	Convergent
Score	32.88 (19.35)	35.51 (19.53)	0.933	3.25%	−0.128	0.950 (0.913‒0.971)	p 0.608 (*R* 0.042)

**Table 3:** Convergent validity analysis: FROI, SCHNOS, and SF-36 domains.

		Physical functioning	Role limitations (physical)	Pain	Role limitations (emotional)	Vitality	Social functioning	Mental health	General health perceptions	SCHNOS Score
FROI Score	*R*	−0.131	−0.022	−0.187	0.038	−0.022	0.066	−0.009	0.042	−0.102
	p	0.401	0.891	0.230	0.808	0.888	0.676	0.954	0.788	0.515

## Discussion

Quality of life assessment has become a widely used practice in healthcare to monitor patient progress and evaluate the success of surgical interventions. In the context of rhinoseptoplasty, the application of validated and culturally adapted instruments for the Portuguese language is essential for the correct interpretation of results, allowing for a more accurate analysis of both functional and aesthetic outcomes. Among the available questionnaires, the Functional Rhinoplasty Outcome Inventory-17 (FROI-17) stands out as a specific instrument for assessing nasal function-related quality of life, considering both functional and aesthetic aspects. The translation, cultural adaptation, and validation of the FROI-17 for Brazilian Portuguese, as carried out in this study, enable a better understanding of the impact of rhinoseptoplasty on adult patients.

Previous studies have demonstrated that the FROI-17 is widely used to measure the functional outcomes of rhinoplasty, being considered a robust method to evaluate nasal symptoms and quality of life. Wähmann et al.^7^ emphasize the importance of standardized instruments like the FROI-17 for clinical practice, as they allow for the identification of significant changes in patients' perception regarding nasal function and aesthetics after surgery. Moreover, as evidenced by Bulut et al.,^5^ the application of the FROI-17 in patients undergoing septorhinoplasty demonstrated significant improvements in postoperative scores, reinforcing its ability to detect meaningful functional changes.

Another relevant aspect is the influence of demographic factors on FROI-17 scores. Plath et al.^6^ identified that age and sex significantly affect FROI-17 scores and patients' self-confidence levels. However, our findings contrast with these results, as no statistically significant differences were observed between sexes concerning overall FROI-17 scores (p = 0.220). This discrepancy may be associated with the age difference between the studied populations, considering that Plath et al. evaluated individuals with a higher average age compared to our sample. Additionally, we observed significant differences in nasal symptoms between genders, with women presenting a lower median score but with greater variability (0–29 for women and 0–27 for men). This difference may be related to the perception of nasal symptoms, which may be more pronounced in women compared to men, as previously reported in studies on satisfaction in facial aesthetic procedures.

In the clinical context, the FROI-17 proves to be a highly useful tool for identifying patients who may benefit from a more targeted surgical approach, enabling a more individualized planning process. The analysis of its psychometric properties demonstrated that the instrument has high internal consistency (Cronbach's α = 0.93) and excellent reproducibility (ICC = 0.95), reinforcing its reliability for measuring significant postoperative changes.

Another relevant aspect was the analysis of agreement and measurement error, which showed values within acceptable limits for clinical assessment instruments. The Standard Error of Measurement (SEM) and the Minimal Detectable Difference (MDD) provided important parameters for interpreting clinically significant changes in patients' scores. These indicators are essential for differentiating real changes from random variations in responses.

Thus, the Brazilian version of the FROI-17 significantly contributes to clinical practice by enabling an objective and standardized assessment of surgical outcomes in rhinoseptoplasty. Moreover, its combined use with already validated instruments, such as the SF-36 and SCHNOS, enhances the understanding of both functional and aesthetic impacts on patients' quality of life. The use of a validated and culturally adapted questionnaire for the Brazilian population allows for a more accurate assessment of the impacts of surgical intervention, providing valuable insights for clinical practice and surgical decision-making.

The process of translation, cross-cultural adaptation, and validation of the FROI-17 for the Brazilian context was successful, proving to be a reliable and relevant instrument for assessing quality of life in patients undergoing rhinoseptoplasty. Future studies may explore the use of the FROI-17 in different population subgroups, as well as its application in long-term evaluations of surgical outcomes.

## Conclusion

The Brazilian Portuguese version of the FROI-17 proved to be a valid and reliable instrument for assessing functional and aesthetic outcomes in patients undergoing rhinoseptoplasty. It enables objective, standardized evaluation of surgical results and enhances clinical decision-making in otorhinolaryngology.

## ORCID ID

Maria Eduarda Malosa Jorge: 0009-0001-9547-609X

Ana Caroline Soares Silva: 0009-0001-9990-8267

Araken Quedas: 0009-0003-0007-2813

Daniela Malheiros: 0009-0000-9311-2463

Guilherme Mororo: 0000-0002-5799-6510

Ingo Baumann: 0000-0001-6945-2601

Eduardo Landini Lutaifi Dolci: 0000-0001-6211-9451

Cinthya Cosme Gutierrez Duran: 0000-0001-9055-1524

## Data availability statement

The authors declare that all data are available in repository.

## Declaration of competing interest

The authors declare no conflicts of interest.
