# Supplementary material for: Translation, cross-cultural adaptation, and validation into Brazilian Portuguese of the Functional Rhinoplasty Outcome Inventory-17 (FROI-17) for use in patients undergoing Functional Rhinoplasty
Source: Braz J Otorhinolaryngol. 2026 May 23;92(5):101773. doi: 10.1016/j.bjorl.2026.101773 (PMC13223699; doi:10.1016/j.bjorl.2026.101773)
Supplement: Supplementary file 1 [file mmc1.docx]

| **Para avaliar o quanto os sintomas individuais têm um impacto, circule o ponto correspondente a cada pergunta** | | **Sem problema** | **Problema muito leve** | **Problema Leve** | **Problema moderado** | **Grande problema** | **Problema por pior que pode ter** |
| --- | --- | --- | --- | --- | --- | --- | --- |
| 1 | Obstrução nasal | 0 | 1 | 2 | 3 | 4 | 5 |
| 2 | Nariz constantemente escorrendo | 0 | 1 | 2 | 3 | 4 | 5 |
| 3 | As secreções fluem para a garganta | 0 | 1 | 2 | 3 | 4 | 5 |
| 4 | Secreção nasal mucosa espessa | 0 | 1 | 2 | 3 | 4 | 5 |
| 5 | Garganta seca | 0 | 1 | 2 | 3 | 4 | 5 |
| 6 | Sensação de pressão nos ouvidos | 0 | 1 | 2 | 3 | 4 | 5 |
| 7 | Comprometimento olfatório | 0 | 1 | 2 | 3 | 4 | 5 |
| 8 | Dificuldade para adormecer | 0 | 1 | 2 | 3 | 4 | 5 |
| 9 | Despertar noturno | 0 | 1 | 2 | 3 | 4 | 5 |
| 10 | Sonolência diurna | 0 | 1 | 2 | 3 | 4 | 5 |
| 11 | Baixa concentração | 0 | 1 | 2 | 3 | 4 | 5 |
| 12 | Diminuição da energia | 0 | 1 | 2 | 3 | 4 | 5 |
| 13 | Irritabilidade | 0 | 1 | 2 | 3 | 4 | 5 |
| 14 | Depressão | 0 | 1 | 2 | 3 | 4 | 5 |
| 15 | Baixa autoestima | 0 | 1 | 2 | 3 | 4 | 5 |
| 16 | Formato do meu nariz, estou envergonhado | 0 | 1 | 2 | 3 | 4 | 5 |
| 17 | Efeitos adversos gerais do nariz (a forma e função) | 0 | 1 | 2 | 3 | 4 | 5 |
